# Internet-based self-help smoking cessation and alcohol moderation interventions for cancer survivors: a study protocol of two RCTs

**DOI:** 10.1186/s12885-018-4206-z

**Published:** 2018-04-02

**Authors:** Ajla Mujcic, Matthijs Blankers, Brigitte Boon, Rutger Engels, Margriet van Laar

**Affiliations:** 1 Trimbos-institute, Netherlands Institute of Mental Health and Addiction, Da Costakade 45, 3521 VS Utrecht, The Netherlands; 2 Utrecht University, Domplein 29, 3512 JE Utrecht, The Netherlands; 3 Arkin Mental Health Care, Klaprozenweg 111, 1033 NN Amsterdam, The Netherlands; 4 Academy het Dorp & Siza, Kemperbergerweg 139E, 6816 RP Arnhem, The Netherlands; 5 Academic Medical Center, Department of Psychiatry, University of Amsterdam, Meibergdreef 9, 1105 AZ Amsterdam, The Netherlands

**Keywords:** Cancer survivors, Psychosocial oncology, Lifestyle behaviours, Smoking, Alcohol, eHealth

## Abstract

**Background:**

Brief interventions for smoking cessation and alcohol moderation may contribute considerably to the prevention of cancer among populations at risk, such as cancer survivors, in addition to improving their general wellbeing. There is accumulating evidence for the effectiveness of internet-based brief health behaviour interventions. The objective of this study is to assess the effectiveness, patient-level cost-effectiveness and cost-utility of two new online theory-based self-help interventions among adult cancer survivors in the Netherlands. One of the interventions focuses on alcohol moderation, the other on smoking cessation. Both interventions are tailored to cancer survivors.

**Methods:**

Effectiveness will be assessed in two separate, nearly identical 2-armed RCTs: alcohol moderation (AM RCT) and smoking cessation (SC RCT). Participants are randomly allocated to either the intervention groups or the control groups. In the intervention groups, participants have access to one of the newly developed interventions. In the control groups, participants receive an online static information brochure on alcohol (AM RCT) or smoking (SC RCT). Main study outcome parameters are the number of drinks post-randomisation (AM RCT) and tobacco abstinence (SC RCT). In addition, cost-data and possible effect moderators and mediators will be assessed. Both treatments are internet-based minimally guided self-help interventions: MyCourse – Moderate Drinking (in Dutch: MijnKoers – Minderen met Drinken) and MyCourse – Quit Smoking (MijnKoers – Stoppen met Roken). They are based on cognitive behaviour therapy (CBT), motivational interviewing (MI) and acceptance and commitment therapy (ACT). Both interventions are optimized in collaboration with the target population of cancer survivors in focus groups and interviews, and in collaboration with several experts on eHealth, smoking cessation, alcohol misuse and cancer survivorship.

**Discussion:**

The present study will add to scientific knowledge on the (cost-)effectiveness of internet-based self-help interventions to aid in smoking cessation or alcohol moderation, working mechanisms and impact on quality of life of cancer survivors. If found effective, these interventions can contribute to providing evidence-based psychosocial oncology care to a growing population of cancer survivors.

**Trial registration:**

Trials are prospectively registered in The Netherlands Trial Register (NTR): NTR6011 (SC RCT), NTR6010 (AM RCT) on 1 September 2016.

## Background

In the last decades, milestones have been reached in fighting cancer. For many types of cancer, survival rates after diagnosis have improved. This has led to a lower mortality rate and a larger population of cancer survivors for many types of cancers, especially in developed countries with aging populations, such as the Netherlands [1, 2]. In 2016, 559,170 men and women in the Netherlands had been diagnosed with cancer in the previous 10 years [3] – which is our working definition of a cancer survivor. Projections indicate that this number will rise to 660,000 cancer survivors in the Netherlands in 2020 (with about equal proportions of men and women) [1]. These people are at an increased risk of facing a recurrence of cancer or second cancers [4].

One of the reasons that nowadays second cancers occur more frequently are adverse effects of cancer therapies. However, it is estimated that less than 10% of second cancers occurring among adults can be attributed to radiotherapy [5, 6], while the magnitude of the risk attributable to chemotherapy is very much dependent on the actual class of anti-cancer agent used, and the dose [5]. This suggests that other overall cancer risk factors (i.e. genetic susceptibility, age, environmental factors, lifestyle factors, and combinations of these factors) are important contributors to second cancer risk [5–7].

Smoking, excessive alcohol drinking and excessive bodyweight are among the main preventable risk factors for developing (second) cancers [7, 8], with alcohol and tobacco related cancer sites accounting for 35% of all second cancers [9]. Especially the impact of alcohol as a carcinogen is often underestimated, but like tobacco it contributes considerably to the disease burden from cancers (i.e. [8, 10, 11]). Targeting smoking and excessive alcohol use would not only potentially contribute to preventing second cancers, but also to improving cancer survivors’ quality of life [12, 13]. Cancer survivors are recommended a healthy lifestyle, including a sufficient amount of daily physical activity and healthy diet, without smoking and limited or no alcohol use [14].

Smoking cancer survivors constitute a substantial subgroup of 9.3% of all cancer survivors, and 15% for lung cancer survivors [15]. Most of these current smokers (83%) smoked daily, averaging 14.7 cigarettes per day [15–17]. Alcohol use among cancer survivors does not differ from alcohol use among the general population [18], 6% of the Dutch general population [19] drinks more than the maximum amount of alcohol containing drinks to prevent cancer occurrence (1 glass/day for women, 2 glasses/day for men), as at the time recommended by the World Cancer Research Fund (WCRF) [14]. Several studies report similar results for cancer survivors [20–22]. Male, younger aged head and neck cancer survivors seem to be more likely to engage in risky alcohol use [23]. Based on a total of 660,000 cancer survivors by year 2020, these figures (9.3% smoking and 6% excessively drinking) translate to approximately 61,000 smoking and 39,000 excessively drinking Dutch cancer survivors. For Europe it is estimated that in the year 2020, over 4 million new cancers will be identified; the previous figures imply 383,000 smoking and 247,000 excessively drinking cancer survivors [24]. These cancer survivors could potentially benefit from tailored, evidence-based support to help them quit smoking or limit their alcohol intake.

Based on recent systematic reviews of RCTs among people who smoke or drink excessively, there is accumulating evidence that guided and unguided internet-based interventions for alcohol moderation (AM) [25] and smoking cessation (SC) [26] can be effective, but also leave room for improvement as effect sizes tend to be small. For internet-based alcohol interventions in particular, a recently published meta-analysis, including a total of 16 randomised controlled trials (with 23 comparisons and 5612 participants), showed a small but significant overall effect size in favour of internet interventions, compared to waitlisted participants, information brochures, or assessment only, but this effect is not sustained after 12 months [25]. A paper integrating all recent reviews on this topic came to similar conclusions [27]. In a Cochrane review on internet-based SC interventions, 28 randomised or quasi-randomised trials were included, yielding data from over 45,000 participants. Results were mixed. All in all, internet-based interventions for SC show some positive results for the general population, but leave room for improvement beyond the standard CBT-based internet interventions [26]. A rather new promising therapeutic approach is Acceptance and Commitment Therapy (ACT), part of third-wave CBT. Both therapeutic approaches have shaped the interventions described in this paper, which will be elaborated further in the intervention descriptions.

Although the potential of internet-based interventions to improve lifestyle factors among cancer survivors is recognized [28], most of currently reported interventions target diet and physical exercise [29]. In a recent study, Bantum et al. [30] tested the effectiveness of a six-week Web-based multiple health behaviour change program for adult survivors compared to a waitlist condition in an RCT (*n* = 352). Cancer survivors were eligible if they had completed their primary cancer treatment from 4 weeks to 5 years before enrolment. The web-based intervention positively impacted reduction of insomnia and frequency of exercise [30]. Further, a web-based, tailored SC program for young adult and childhood cancer survivors yielded positive SC outcomes at 15 months post-randomisation in an RCT comparing web and print-based materials. Both versions yielded quit rates (*n* = 374, web-based: 16.5%, print-based: 15.5%) that are similar to the intensive telephone counselling treatment they were based on (15% at 12 months post-randomisation) [31].

Thus far, specific AM and SC internet-based intervention RCTs have not been focused specifically on cancer survivors, with a few exceptions [31, 32]. There is a lack of knowledge on what results in terms of effectiveness and cost-effectiveness could be obtained when existing internet interventions for smoking and alcohol would be tailored to cancer survivors. Based on (Cochrane) reviews on other lifestyle interventions, positive outcomes can be expected [28, 30, 33–35]. Furthermore, time of diagnosis is referred to as a ‘teachable moment’ [36, 37]; cancer diagnosis might trigger cancer survivors and possibly their family members [37] to adapt a more healthy lifestyle and may thus be a good moment to introduce health promotion programs.

The objective of the two RCTs presented in the current study protocol is to test the effectiveness of two newly developed online interventions to reduce alcohol use or tobacco smoking in samples of excessive drinking or smoking cancer survivors.

## Methods

### Aims and hypotheses

The overall aim of the study is to examine the effectiveness and cost-effectiveness of two internet-based interventions for cancer survivors. One intervention focuses on alcohol moderation (AM), the other intervention on smoking cessation (SC). Both interventions will be compared to information-only control groups (CTRL) in a randomised controlled trial (RCT).

It is hypothesized that:

- The experimental internet-based AM intervention will reduce alcohol use more than CTRL, 6 months post-randomisation.
- The experimental internet-based AM intervention will show favourable cost-effectiveness (cost per quality-adjusted life year < 20,000 euro) compared to CTRL.
- The experimental internet-based SC intervention will lead to a higher quit rate than CTRL, 6 months post-randomisation.
- The experimental internet-based SC intervention will show favourable cost-effectiveness (cost per quality-adjusted life year < 20,000 euro) compared to CTRL.

### Study design

Two separate two-arm RCTs will be carried out (alcohol moderation (AM RCT) and smoking cessation (SC RCT)), each with a follow-up duration of 12 months in an online context. Study design, procedures and measurement instruments of the two RCTs are the same – the main difference is the aim of the intervention (either alcohol moderation or smoking cessation). The RCTs have been designed in line with the CONSORT statement [38]. Both studies are registered in the Dutch Trial Register; identifiers: NTR6010 (AM RCT) and NTR6011 (SC RCT). Ethical approval to carry out the studies was obtained from an accredited medical research and ethics committee in the Netherlands (Toetsingscommissie Wetenschappelijk Onderzoek Rotterdam e.o. NL55921.101.16).

### Study procedure

Applicants meeting inclusion criteria will be informed on the conditions of participation. If they would like to participate, participants are asked for necessary personal data. An invitation email will be sent to them containing the informed consent form, all relevant patient information and a link to register. From this moment on, they have up to 30 days to decide if they want to participate or not. During these 30 days, they can contact a member of the research team responsible for the inclusion process by phone or email or face-to-face for questions regarding the study and interventions. They can also contact an independent physician during these 30 days. After their signed informed consent has been received digitally they are invited to the baseline questionnaire. After they have completed the baseline measurement, randomisation takes place. Depending on the outcome, participants are allocated to one of the two trial arms (active self-help internet-based intervention or passive internet-based information brochure). Participants receive a confirmation email containing a username and instructions how to log in.

**Fig. 1:**
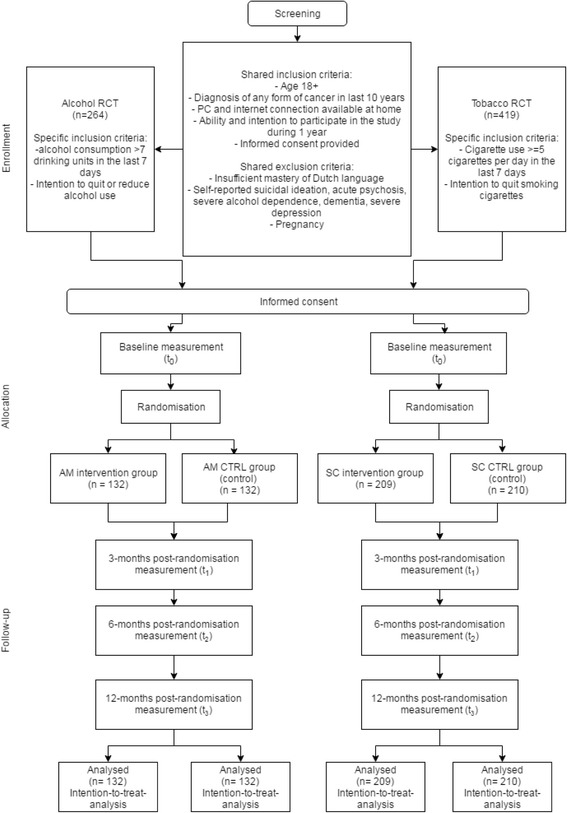
Flowchart of participant movement in the AM RCT and SC RCT

Follow-up measurement waves will take place at 3, 6 (primary endpoint), and 12 months post-randomisation (Fig. 1). At each measurement point, participants receive an email including a link to the online questionnaires. Non-respondents receive three reminder emails and are subsequently contacted by telephone in case of continued non-response. As responses are collected online, all data are automatically validated (range checks etc.) on the client side, and after validation stored in a secured server-based database. All data transferred between client and server are encrypted using the Transport Layer Security cryptographic protocol.

### Randomisation

After completing the baseline measurement, participants will be allocated to the two trial arms in a 1:1 ratio. As the number of participants we aim to include in each trial arm is not very large, random variation in baseline characteristics could reduce trial arm equivalence. Therefore, allocation through adaptive randomisation (Minimisation [39, 40]) will be used to balance trial arms with regard to age, sex, and education level. Adaptive randomisation implies that the randomisation sequence is not a priori known but is based on the variance in variables that need to be balanced over the trial arms. If imbalance between the trial arms in age, sex or education occurs, the probability of allocation to the trial arm that minimizes this imbalance is increased to 0.67 (instead of 0.5). The randomisation procedure is automatized and performed by triggering a server-sided PHP script using a Mersenne twister random number generator, immediately after the participant has completed the baseline measurement. After randomisation, the participant is informed about the outcome of the randomisation via an automated email, and assigned to one of the two conditions via an automated server-sided computer script. After this assignment, the researchers are informed about the outcome and assignment of the participant via an automated registration in the trial management database. As this is an open label RCT, the participants nor the researchers are blinded regarding the allocated conditions.

### Participants

#### Recruitment

The population base from which the subjects will be drawn are Dutch adult cancer survivors, meeting in/exclusion criteria. A website is created, containing information on the study and the possibility to enrol as a participant. Collaboration with Dutch patient organizations is sought and all (social) media channels available will be utilized to ensure recruitment of the planned number of participants. Other recruitment strategies will include: reaching out to smoking cessation clinics, oncology nurses and meeting centers for cancer survivors, online advertisements on (health-related) websites, targeted facebook and search engine campaigns, and advertisements in newspapers and magazines relevant to the target group.

#### In- and exclusion criteria

All potential participants fill out an online screening questionnaire to determine whether they fulfil all inclusion criteria and none of the exclusion criteria. There are four possible outcomes: 1) inclusion criteria are not met and applicants cannot participate in the study; 2) inclusion criteria for the alcohol RCT are met and they are invited to participate in the alcohol RCT, 3) inclusion criteria for the tobacco RCT are met and they are invited to participate in the tobacco RCT, 4) inclusion criteria for both RCTs are met and participants can choose in which one of the two RCTs they want to participate (they cannot participate in both RCTs). Participants who do not fulfil criteria for inclusion will be provided with links to websites with further alcohol/smoking information and help.

Shared inclusion criteria (for both RCTs):

- Age 18+
- Diagnosis of any form of cancer in the last 10 years
- PC and internet connection available at home
- Ability and intention to participate in the study and the intervention during the period of one year
- Informed consent provided

Additional inclusion criteria for the AM RCT only:

- Alcohol consumption of > 7 standard drinking units (10 g of ethanol) in the last 7 days
- Intention to reduce or quit alcohol use as assessed by one item from the screening questionnaire

Additional inclusion criteria for the SC RCT only:

- Cigarette use of > = 5 cigarettes per day in the last 7 days
- Intention to quit smoking cigarettes as assessed by one item from the screening questionnaire

Shared exclusion criteria (for both RCTs):

- Insufficient mastery of Dutch language
- Self-reported suicidal ideation, acute psychosis, severe alcohol dependence, dementia, severe depression
- Self-reported pregnancy

### Sample size

For both the AM RCT and SC RCT, conventional power (1-beta = .80) and levels of statistical significance (alpha = .05) are chosen. For both trials, the primary outcome data is collected at 6 months post-randomisation.

For the AM RCT, the primary outcome variable is based on the 7-day TLFB alcohol measurement, 6 months post-randomisation. Based on the average of 2 previous RCTs on very similar self-help interventions in the Netherlands versus a control condition (see Riper 2008 and Blankers 2011 in [25]), a Cohen’s d effect size of d = 0.40 is expected. Using the power calculation package “pwr” [41] for R 3.0.1 [41], d = .40 translates into a minimum net sample size of 2 × 99 participants in case of 2-sided testing, or 2 X 78 participants in case of 1-sided testing. Assuming a maximum of 25% non-response at 6 months follow-up, we intend to include 99 × 2 x (100/(100–25)) = 264 (or 208 for 1-sided testing) participants in the alcohol RCT. In case the drop-out rate is lower than 25%, power will be somewhat higher than we anticipate in this calculation, i.e. in that case we are more likely to find a true effect.

For the SC RCT, the primary outcome variable is based on the 7-day TLFB smoking measurement: self-reported abstinence in the last 7 days prior to the primary measurement point, 6 months post-randomisation. Based on a study by Duffy et al. amongst cancer survivors [12], a quit-rate of 30% in the active smoking cessation intervention group, vs 15% in the smoking cessation control group can be expected. This translates into a relative risk (RR) of 2.14, which is comparable to the RR estimate in a recent Cochrane review (RR = 2.05) [26]. Based on a pilot trial of an ACT smoking cessation internet intervention [42], a 23% quit rate in the experimental arm vs a 10% quit rate in the control arm can be expected (RR = 2.20), at the 3 months follow-up. Based on the average of these RRs, a RR = 2.1 is expected. This translates into a 21% quit rate in the experimental condition, assuming a 10% quit rate in the control condition at 6 months post-randomisation. Using “pwr” [43] for R 3.0.1 [41], a 21% quit-rate vs 10% quit-rate translates into a net sample size of 2 × 157 participants, based on 2-sided tests (2 X 124 for 1-sided tests). Assuming 25% non-response at 6 months follow-up, we need 157 × 2 x (100/(100–25)) = 419 participants in the SC RCT for 2-sided tests, and 331 for 1-sided tests).

The described power analyses are conservative. We may perform half-way post-hoc power analyses for both RCTs, based on those we will evaluate our assumptions underlying these power calculations and may adjust the sample sizes if necessary. We might for example not fully compensate the expected 25% drop-out, as drop-out rates might be lower and we will perform intention-to-treat analyses including multiple imputation, which partially recovers power. One-sided testing is mentioned because it is very plausible that the interventions will have a positive effect on SC and AM [25, 26, 42]. Furthermore, within the above mentioned calculation, clustering of measurements (baseline, 3-, 6- and 12-months) within participants and related covariance has not been taken into consideration. Accounting for this could also change required sample sizes.

### Conditions

#### MyCourse – Quit smoking and MyCourse – Moderate drinking

**Fig. 2:**
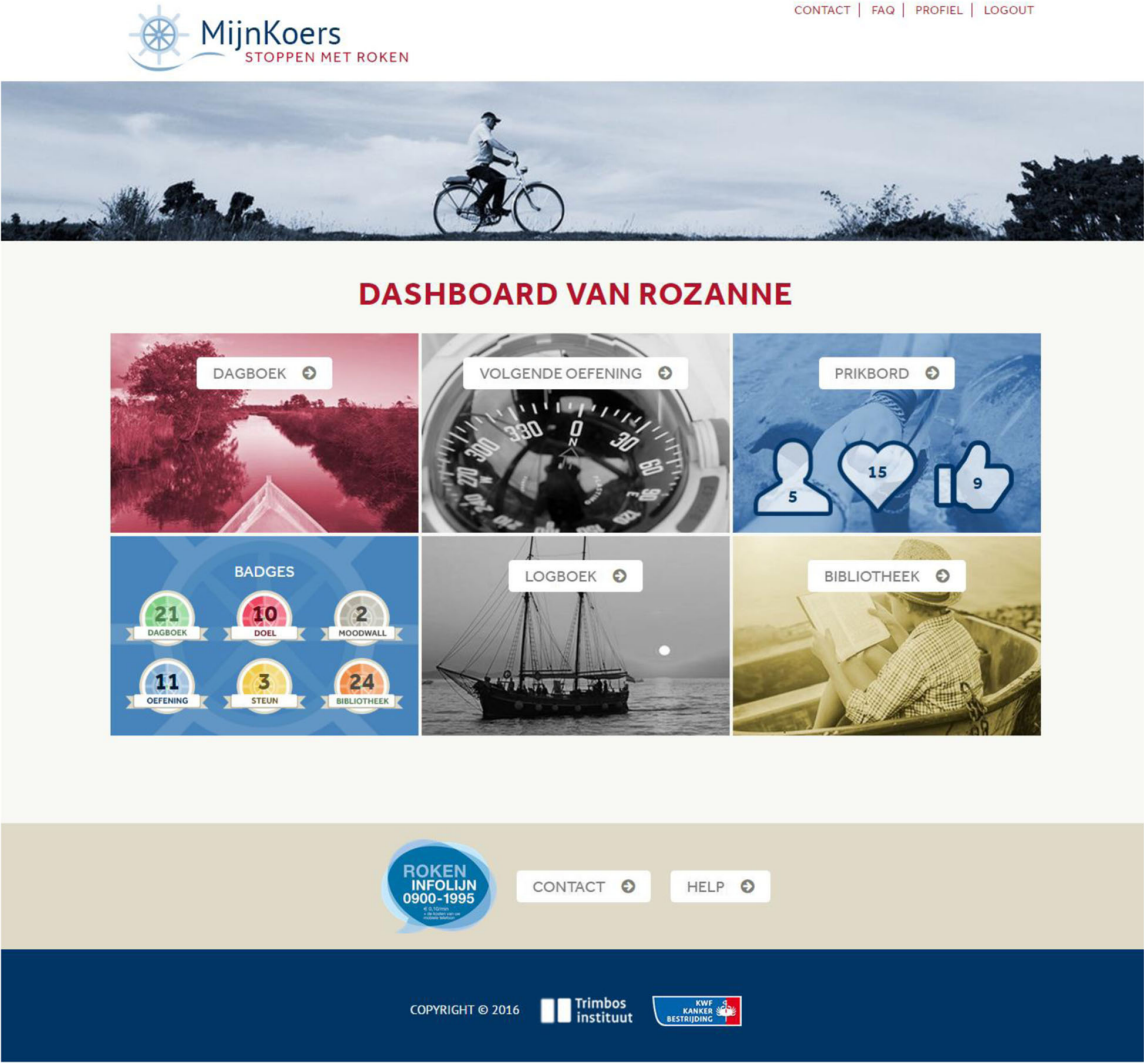
Main page of new online intervention MyCourse – Quit Smoking (in Dutch)

Both programs are online lifestyle interventions optimized for cancer survivors: MyCourse – Moderate Drinking (in Dutch: MijnKoers – Minderen met Drinken) and MyCourse – Quit Smoking (MijnKoers – Stoppen met Roken) (see Fig. 2). The interventions are developed and tailored in collaboration with the target population of cancer survivors in focus groups and interviews, and in collaboration with several experts on eHealth, smoking cessation, alcohol misuse and cancer survivors.

#### Therapeutic approaches: CBT, ACT and MI

Both interventions are based on cognitive behavioural therapy (CBT), acceptance and commitment therapy (ACT) and motivational interviewing (MI) techniques.

CBT has been well-established as an effective therapeutic approach for treating excessive alcohol use and aiding in smoking cessation in web-based programs [25, 26, 44]. It aids participants in understanding connections between cognitions, context and behaviour and hands them skills to cope with cognitions and situations that elicit unwanted behaviour. MI techniques help participants identify their ambivalences towards quitting smoking or moderating alcohol use, and help solve them [45]. MI has demonstrated a modestly significant effect in efficacy studies on smoking cessation, (RR 1.27; 95% CI 1.14 to 1.42) [45] and excessive alcohol drinking (d 0.40; 95% CI 0.17 to 0.70) [46]. National CBT- and MI-based treatment protocols were used to shape this part of the interventions [47, 48].

ACT is an emerging theory-based treatment paradigm that has demonstrated feasibility and efficacy in SC treatment in several studies and in a variety of modalities (face-to-face, telephone-based or web-based) [42, 49–51]. Regarding AM, a pilot trial found ACT-based group therapy for alcohol disorder and comorbid affective disorder effective [52]. Including the treatment of other substance use disorders (i.e. opioids, amphetamines, polydrug use) ACT shows favourable efficacy compared to other active treatment conditions (e.g. CBT and 12-step programs) and sustains the effects for a longer follow-up period [52, 53]. Acceptance in ACT stands for allowing intense physical sensations, cognitions, and emotions which may trigger drinking (AM RCT) and smoking (SC RCT) to come and go, without trying to control them; commitment stands for keeping in mind what is important to individuals (values) in order to guide action plans (stopping smoking) [42]. Specifically, ACT focuses on identifying thoughts, feelings, and physical sensations that trigger the target behaviour [42]. Unlike traditional CBT, ACT does not teach methods to avoid or control these triggers, but it focuses on changing one’s relationship with them by allowing them to be present without acting on them [42, 49, 54].

#### Optimization for cancer survivors

Patients participated throughout the development process. This resulted in all exercises being written in such a way as to better suit the needs of the population of cancer survivors. Information on short-term benefits is placed more prominently within the informative texts. Positive reinforcement is emphasized and effectuated in multiple ways, including badges and verbal reinforcements within the exercises. Support from the participant’s own social network was deemed highly important in the focus groups, so several exercises include a feature that enables quick, easy and personalized updates by sending a direct email to a friend, partner or relative. In addition, information regarding alcohol/tobacco and cancer interactions is included in the intervention. Because cancer survivors constitute a generally older age group, design has been simplified as much as possible. Clear instructions are given on every page, always including a help button, sharp contrasts ease reading.

#### Intervention flow

**Fig. 3:**
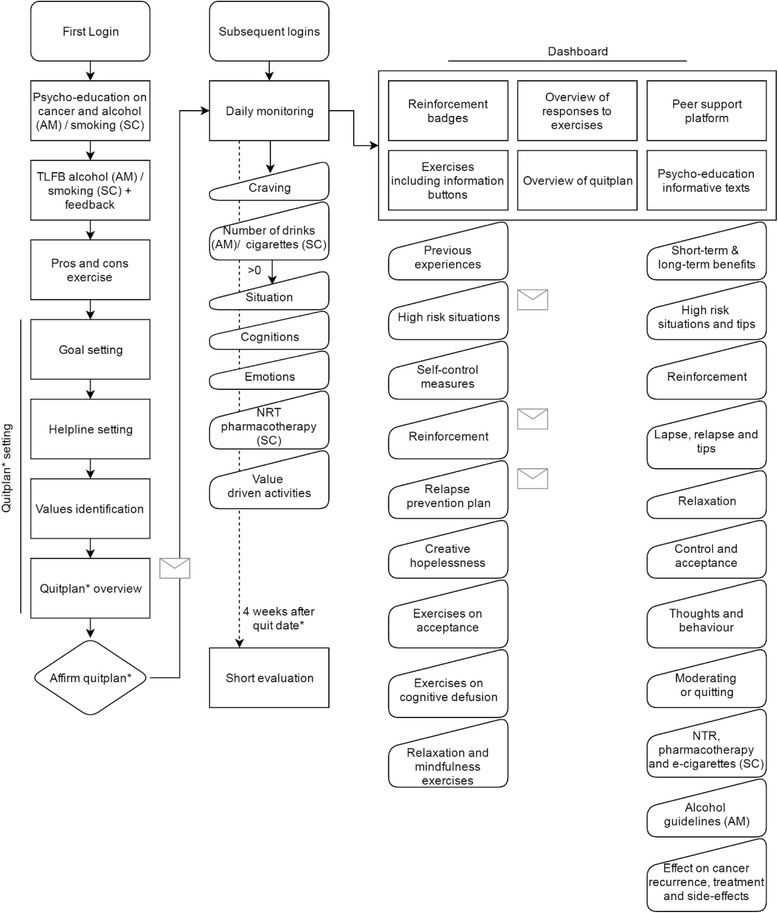
Structure of online interventions MyCourse – Quit Smoking (SC) and MyCourse – Moderate Drinking (AM) *Legend:* Mail icons demonstrate semi-automatized email options to participants’ own social network. * Moderation plan/moderation date is possible in AM only

Both interventions are accessible through PC, tablet/iPad and smartphone. Length of the two interventions is equal. Participants are advised to use the intervention for 4 weeks after their set quit/moderation date, but they are free to quit whenever they want. After 4 weeks participants evaluate their goal achievement in the intervention. After this short evaluation all intervention components will remain available for at least 12 months. Table 1 shows descriptions of the main intervention elements, an overview of movement through these elements is shown in Fig. 3.

**Table 1:** Main elements within new online interventions MyCourse – Quit Smoking and MyCourse – Moderate Drinking

Main elements	Description
Goal setting	Participants set a quit plan (SC and AM RCT) or moderation plan (only AM RCT) including a quit date
Goal monitoring	Every day participants monitor their drinking or smoking behaviour, mood, cognitions, and contextual cues which have led them to drink or smoke. Feedback is provided in a personalized graph
Exercises based on CBT and ACT	Exercises help identify high risk situations for excessive drinking/smoking and self-management strategies.
	ACT-exercises help accept difficult feelings while keeping focused on the behaviour goal and help exercise self-compassion to prevent relapse
Psycho-education	Effect of alcohol/tobacco on cancer, cancer treatment and life after cancer
Reminders	Several automated email reminders to regularly log on, monitor behaviour and finish all exercises
Peer support platform	A moderated bulletin board, focused on sharing tips and experiences
Social support from social network	Semi-automated email functions throughout the program to send personalized, informing emails to a trusted person

At the first login, participants are prepared for action using motivational interviewing-based techniques. Advantages and disadvantages of drinking (in AM RCT) or smoking (in SC RCT) and moderation/quitting are assessed. Next, ACT’s core process of committed action is targeted by having users apply their core values guiding quitting towards a personalized quit plan. Only for AM RCT: If moderation is chosen as a goal, the maximum amount of alcohol consumed per day and per week is set. The participant is also asked to set a date to start working towards the new drinking (AM RCT) or smoking cessation (SC RCT) goals. The quit date is to be set within 1 week upon logging in for the first time.

The next stage is the behaviour change phase, based on CBT. Here, participants are asked to monitor their drinking (AM RCT) or smoking (SC RCT), their mood, their cognitions, and contextual cues which have led them to drink (AM RCT) or smoke (SC RCT). Based on the monitoring data they provide, feedback is generated in the form of a graph to show progress towards their goals at a glance. This monitoring screen is shown first upon subsequent logins. In line with ACT, participants also monitor whether they have acted on their values each day. This is phrased as having engaged in a positive activity, where participants can choose from the values they have selected when setting their Quit/Moderation plan.

A personalized dashboard shows the different intervention components. Exercises are provided to help participants gain better insight into their drinking behaviour, and ways to handle cravings and high risk situations. In the final stage, participants learn how to manage relapse, and how to maintain behaviour change. ACT’s core processes of acceptance (preparedness to experience feelings or sensations), being present (staying connected with the here-and-now), cognitive reflection (watching the process of thinking), and self-as-context (awareness of the difference between one’s self and one’s thoughts) are targeted through a series of exercises designed to enhance these skills. Participants are invited to use these skills when they have urges, experience withdrawal symptoms or lapses [42].

On the peer support platform participants can provide and receive support from other participants during the intervention. Additionally they are encouraged to seek support from their own social network. Throughout the program, participants have the ability to share some of their answers with their partner or someone else who provides them support via semi-automatized emails. For example, they can share their quit plan or their high risk situations, thus helping their supportive social network in helping them by providing them with key information.

At any time, participants have quick access to an overview of their quit plan and finished exercises. To enhance adherence, emailed reminders are sent regularly after the participant has not logged in for several days, has not registered their drinking or smoking behaviour, or has not completed all exercises.

Throughout the interventions a sea faring ship is used as a metaphor. This is a means to help participants understand and experience the gist of ACT principles and foster continuity throughout the different intervention elements. Metaphors are used in ACT to loosen the grip of our cognitive thoughts on our feeling of self and on our behaviour. Using metaphors can circumvent the inclination to verbally protest against, for example, the exercise that explains how trying to exert control all the time will most likely not benefit you [55].

### Control conditions

The two control-condition interventions provide plain information on risks of alcohol (AM RCT) or smoking (SC RCT) in general and information specifically relevant for cancer survivors. Tips on how to reduce or quit alcohol use or quit smoking are also provided, but do not include the interactive elements that are part of the self-help interventions. Participants in the control groups can access the information page as often as they want by logging in on the website. However, the information on the information pages is static, does not change over time, and is not tailored to the individual participant. After completion of the study, 12 months post-randomisation, these participants are also provided with access to the self-help interventions. All participants are free to seek additional support if needed, use of additional support will be assessed in follow-up measures.

### Outcome measures

An overview of all measures and their measuring points is given in Table 2.

**Table 2:** Schematic representation of outcome measures and measurement waves

Assessments (number of items)	baseline (t_0_)	3-months post-randomisation (t_1_)	6-months post-randomisation (t_2_)*	12-months post-randomisation (t_3_)
AAQ-II (7)	x	x	x	x
AUDIT (10)	x		x	
Knowledge questionnaire (12)	x	x	x	
BSI-18 (22)	x		x	
EQ-5D (5 + 1)	x	x	x	x
Fagerstrom Test for Nicotine Dependence (6)	x	x	x	x
MCSDS (13)	x			
OCDS^1^ (5)	x^1^	x^1^	x^1^	
Perceived partner support (1)	x	x	x	x
Self-efficacy measure (3)	x	x	x	x
SF36 (36)	x	x^a^	x	x^a^
Socio-demographics (24)	x			
TiC-P (31)	x	x	x	x
Timeline-Follow-Back (TLFB) for alcohol consumption (7)	x	x	x	x
Timeline-Follow-Back (TLFB) for tobacco consumption (7)	x	x	x	x
QSU-brief^2^ (10)	x^2^	x^2^	x^2^	
ZUF-8 (8)		x		

^1^= only applied in the AM RCT. ^2^ = only applied in the SC RCT. ^a^ = only 11 items from the SF36 will be administered at 3 and 12 months post-randomisation (necessary to apply the Brazier algorithm). * Primary endpoint for both the AM and SC RCT

### Primary measures

#### Alcohol and tobacco use

Main study parameters are Timeline Follow-Back (TLFB) reports on alcohol use (number of standard drinks) in the 7 days prior to the 6-month post-randomisation measurement wave (AM RCT) [56] and tobacco abstinence measured by TLFB reports on tobacco use (number of cigarettes) in the 7 days prior to the 6-month post-randomisation measurement wave (SC RCT). TLFB reports yield information on frequency as well as patterns of substance use behaviour [57, 58]. Outcomes from online administrated TLFB reports are consistent with face-to-face or telephone administrated TLFB reports [57, 59]. An additional question is sent in the SC RCT about tobacco use in the 14 days prior to 4 weeks after the set quit date, to comply with the Russell Standard and thus make the study better comparable to international smoking cessation studies [60].

### Secondary measures

#### Alcohol and nicotine dependence

Secondary measures include alcohol (AM RCT) or nicotine dependence (SC RCT) as measured by, respectively, the Alcohol Use Disorders Identification Test (AUDIT) [61] and Fagerstrom Test for Nicotine Dependence (FTND) [62]. AUDIT is a 10-item questionnaire on patterns of alcohol use and problems experienced due to alcohol use, to distinguish low-risk from high-risk drinkers. The AUDIT has been validated in 6 countries [61]. FTND is a 6-item questionnaire which has been shown to reliably assess nicotine dependence in a Dutch sample [63].

#### Treatment satisfaction

Treatment satisfaction is measured by Fragebogen zur Messung der Patientenzufriedenheit (ZUF-8) [64], a German version of the CSQ-8 which has shown good psychometric properties (translated in Dutch) [65]. Its eight items are scored on a 4-point scale, without a ‘neutral’ answer option.

### Cost-effectiveness

#### Cost-data and quality of life

Cost-data are measured by the Trimbos/iMTA questionnaire for Costs associated with Psychiatric Illness (TiC-P) [66]. Part 1 measures healthcare consumption through questions on frequency of contact with several health care providers. Part 2 of the TiC-P assesses health related productivity losses. This Dutch questionnaire showed good test-retest reliability and promising construct-validity for items concerning contact with health professionals [67]. Quality of life is assessed by EQ-5D (5 L) [68–71] and MOS SF-36 [72]. Participants state the extent of problems experienced on five dimensions. EQ-5D (5 L) improved discriminatory power compared to EQ-5 L (3 L) and showed good validity across several international patient groups [73]. MOS SF-36 consists of 36 items on 8 dimensions, with higher scores reflecting a higher level of well-being. A Dutch translation showed good psychometric properties [74].

### Mediators and other measures

In addition, several hypothesised intervention effect mediators will be assessed through the following questionnaires: craving using OCDS [75] (AM RCT) and QSU-brief [76, 77] (SC RCT), symptoms of psychopathology BSI-18 [78], experiential avoidance using AAQ-II [79], obtained knowledge on CBT and ACT principles using a 12-item questionnaire [80], a single item on perceived partner support [81], a 3-item questionnaire on self-efficacy to moderate alcohol drinking (AM RCT) or quit smoking (SC RCT) [82, 83], and utilization variables (number of logins, time spent logged in, use of major content elements etc.) [84]. The Marlowe-Crowne Social Desirability Scale (MCSDS) will be included to evaluate the reliability of the self-reported questionnaire data [85]. Collected socio-demographic variables will include age, sex, education, marital status, living situation and cultural background.

Participants will furthermore be asked for permission to access their patient data in the Netherlands Cancer Registry, which is managed by IKNL (Netherlands Comprehensive Cancer Organisation) to obtain reliable data about their disease course. They are also asked for permission to access their healthcare cost-data registered by national statistics organization Statistics Netherlands (Centraal Bureau voor Statistiek (CBS)). Access to these data can be granted or denied by the participants by ticking boxes in the informed consent form.

### Statistical analyses

Outcome data will be analysed using Generalized Linear Mixed Models (GLMM) with log link functions depending on the data types and distributions of the dependent variables (count or continuous data in case of alcohol use, dichotomous data in case of smoking cessation) will be applied to the primary and secondary outcome measures. Missing data will be handled using the multiple imputation package Amelia 2 in the software package R 3.0+, and with at least 1 other package as a comparison. In a benchmark study, this Amelia 2 package outperformed other conventional multiple imputation packages [86]. Analyses will be conducted on the entire randomised sample (i.e. intention to treat) and on the per protocol/ treatment completers sample. All analyses will be carried out using SPSS version 20+ and/or R version 3.0+. Covariates in the model will be the minimised variables (see section on randomisation), variables with a *p* < .05 difference at baseline and the MCSDS. In the above mentioned analyses, clustering of measurements (baseline, 3-, 6- and 12-months) within participants and related covariance has not been taken into consideration.

The economic evaluation will be conducted alongside the randomised trial. The Dutch tariffs (utility weights) [70] and the MVH-A1 tariff by Dolan et al. [87] for the EQ-5D-5 L will be used for computing the QALYs [70]; for the MOS SF-36, the Brazier scoring algorithm (SF-6D) will be used [88]. Using the area under the curve (AUC) method, the periods between the measurement waves will be weighted by the utility of the health state in that period. This allows the computation of quality adjusted life years (QALYs) over the entire trial period. In a similar vein, cumulative costs over the entire follow-up period will be obtained from the cost estimates at the various measurement waves. The cost-effectiveness evaluation will be performed in line with suggestions by Drummond et al. (2015) [89], i.e. in agreement with the intention-to-treat principle, with missing data addressed using imputation. The incremental cost-effectiveness ratio (ICER) will be calculated as follows: ICER = (C_1_–C_2_)/ (E_1_–E_2_), where C are costs, E effects, and subscripts (_1_ and _2_) refer to the two trial arms (experimental/self-help and control/information brochure). Confidence intervals around the ICER will be calculated using a non-parametric bootstrap approach: >2500 non-parametric bootstrapped samples will be extracted from each of the original datasets. For each of these bootstrapped samples, the incremental costs, incremental effects, and the incremental cost-effectiveness ratio (ICER) will be calculated. The resulting >2500 ICERs per dataset will be used for further calculations and will be plotted on a cost-effectiveness plane. In addition, cost-effectiveness acceptability curves (CEACs) will plotted. One-way sensitivity analyses directed at uncertainty in the main cost drivers will be performed to gauge the robustness of our findings.

## Discussion

This paper describes the study protocol for assessing two online self-help interventions aimed at supporting cancer survivors in their attempts to quit smoking or to moderate or quit their alcohol use. Two separate RCTs will determine the effectiveness and cost-effectiveness of MyCourse – Quit Smoking and MyCourse – Moderate Drinking, which have been developed in close collaboration with cancer survivors, and several experts on eHealth, smoking cessation, alcohol misuse and cancer survivorship. Primary outcome measures are smoking abstinence (SC RCT) and number of drinks (AM RCT) at 6 months post-randomisation. Several possible mediators will be examined as well, to gain insight into active mechanisms in online behaviour change interventions.

In this study cancer survivors are described as individuals from the time of diagnosis [90], a definition also adopted by the National Cancer Institute in the USA [91] and the Dutch Cancer Registry (NKR) [92]. Both interventions are offered at any time after diagnosis. Cancer diagnosis is often referred to as a ‘teachable moment’ that could entail increased motivation to adopt health behaviors [36, 93]. But some might argue that only after treatment people can focus their energy on online interventions. Correct timing of these interventions is yet to be studied, although a recent study suggests to offer SC support as soon as possible [94]. Note that cancer survivors involved in the development process were mostly older, hence the interventions might not be specifically tailored to young adult cancer survivors. Online interventions targeting SC in current scientific literature mostly target younger cancer survivors [31, 32]. However, for older cancer survivors online interventions are also likely a suitable mode of delivery, as in 2016, over 89% of Dutch adults aged 45–75 has internet access, over 81% of Dutch adults aged 45–65 uses internet daily, 63% of 65–75 year olds use internet daily, and an additional 13% at least weekly [95]. Searching for health information is among their top internet activities.

The present study will improve the scientific knowledge regarding the effectiveness and cost-effectiveness of internet-based minimally guided self-help interventions to address cigarette use and alcohol misuse among cancer survivors. If found successful, they will be implemented and made available to all interested cancer survivors in the Netherlands. Accordingly, this study contributes to providing evidence-based and sustainable psycho-social oncological care to a growing population. Furthermore, by stimulating health behaviours such as smoking cessation and alcohol moderation, recovery and quality of life after cancer treatment are expected to be improved and the incidence of second cancers is expected to be reduced.

## Abbreviations

AAQ: Acceptance and Action Questionnaire
ACT: Acceptance and commitment therapy
AM: Alcohol moderation
AUC: Area under the curve
AUDIT: Alcohol Use Disorders Identification Test
BSI: Brief Symptom Inventory
CBS: National statistics organization Statistics Netherlands
CBT: Cognitive behavioural therapy
CEAC: Cost-effectiveness acceptability curve
CONSORT: Consolidated Standards of Reporting Trials
CSQ-8: Questionnaire assessing treatment satisfaction
CTRL: Control groups
EQ-5D: EuroQOL 5 dimensional questionnaire to assess quality of life
FTND: Fagerstrom-test for Nicotine Dependence
GLMM: Generalized Linear Mixed Models
ICER: Incremental cost-effectiveness ratio
IKNL: Netherlands Comprehensive Cancer Organisation
MCSDS: Marlowe-Crowne Social Desirability Scale
MI: Motivational interviewing
MOS SF-36: Medical Outcomes Study Short Form Survey assessing quality of life
NKR: Dutch Cancer Registry
OCDS: Obsessive Compulsive Drinking Scale
QALY: Quality adjusted life years
QSU: Questionnaire of smoking urges
RCT: Randomised controlled trial
RR: Relative Risk
SC: Smoking cessation
TiC-P: Trimbos/iMTA questionnaire for Costs associated with Psychiatric Illness
TLFB: Timeline Follow-Back
WCRF: World Cancer Research Fund
ZUF: Patient satisfaction questionnaire
